# Cannabis use and cognition in older adults: Preliminary performance-based neuropsychological test results and directions for future research

**DOI:** 10.1017/S1355617725101203

**Published:** 2025-08

**Authors:** Kyler Mulhauser, Daniel Sullivan, Jessica L. Bair, Anthony N. Correro, Subhamoy Pal, Jonathan Reader, Benjamin M. Hampstead, Bruno Giordani

**Affiliations:** 1 Department of Psychiatry, University of Michigan, Ann Arbor, MI, USA; 2 Michigan Alzheimer’s Disease Center, University of Michigan, Ann Arbor, MI, USA; 3 Mental Health Service, Iowa City VA Healthcare System, Iowa City, Iowa, USA; 4 Department of Neurology, Division of Neuropsychology, Medical College of Wisconsin, Milwaukee, WI, USA; 5 Department of Neurology, University of Michigan, Ann Arbor, MI, USA; 6 Department of Veterans Affairs, Veterans Affairs Ann Arbor Healthcare System, Ann Arbor, MI, USA

**Keywords:** Older adults, cognition, cannabis, neuropsychological assessment, cannabis use frequency, aging and memory

## Abstract

**Objective:**

We evaluated performance-based differences in neuropsychological functioning in older adults (age 65+) across the dementia continuum (cognitively intact, mild cognitive impairment, and dementia) according to recent cannabis use (past six months).

**Method:**

A sample of 540 older adults from a well-characterized observational cohort was included for analysis. Participants completed a standardized questionnaire assessing cannabis use in the six months prior to the study visit and completed a comprehensive neuropsychological assessment. We used traditional cross-sectional analyses (multivariate, univariate) alongside causal inference techniques (propensity score matching [PSM]) to evaluate group differences according to recent cannabis use status. We also examined whether cannabis-related problem severity, a risk factor for cannabis use disorder (CUD), was associated with cognitive outcomes among those reporting recent cannabis use.

**Results:**

Approximately 11% of participants reported using cannabis in the prior six months, with the median user consuming cannabis two to four times per month. Participants with recent cannabis use performed similarly across all five domains of neuropsychological functioning compared to those with no cannabis use. Among older adults reporting recent cannabis use, those with elevated risk for CUD demonstrated lower memory performance.

**Conclusions:**

These preliminary results are broadly consistent with other findings indicating that low-frequency cannabis use among older adults, including those along the dementia continuum, is generally well tolerated from a cognitive perspective. However, among older adults who used cannabis, elevated symptoms of CUD may negatively impact memory performance. Future research should explore how variations in cannabis use patterns, individual characteristics, and clinical phenotypes influence cognitive outcomes.

## Statement of Research Significance

### Research Question(s) or Topic(s):

This study investigated whether older adults who use cannabis perform differently on neuropsychological tests compared to those who do not. **Main Findings:** Older adults who reported using cannabis in the past six months showed no differences in cognitive test performance compared to non-users across five cognitive domains. Among older adults reporting recent cannabis use, greater severity of cannabis-related problems was associated with lower memory performance. **Study Contributions:** This study expands the research base on cannabis use and cognition in older adults. Specifically, this study used a comprehensive battery of neuropsychological tests to evaluate for cognitive differences in older adults according to recent cannabis use status and risk factors associated with cannabis use disorder. Consistent with other research, these preliminary findings suggest that low-frequency cannabis use is generally well tolerated, although hazardous/problematic cannabis use behaviors were associated with lower memory performance.

## Introduction

Older adults (age 65 and older) are using cannabis at unprecedented rates, representing the fastest growing age segment among both new cannabis users and daily cannabis consumers (Han et al. 2017; Han & Palamar, 2018, 2020; Khoury et al. 2022; Statistics Canada, 2019; Statistics Canada & Rotermann, 2020). Approximately 2 – 7% of older adults in North America report using cannabis in the past month, with 2019 estimates revealing that 27% of older adults using cannabis were first-time users (Jeffers et al. 2021; Maxwell et al. 2021; Statistics Canada, 2019). Older adults who consume cannabis now use at rates comparable to that of younger-aged peers, with approximately 60% reporting frequent or daily use (Jeffers et al. 2021). Among older adults who use cannabis, 75% do so for medical purposes – primarily symptom management for pain, sleep, and mood – although the reported therapeutic benefits and harms of cannabis use are mixed (Briscoe & Casarett, 2018; Minerbi et al. 2019; Tumati et al. 2022; Wolfe et al. 2023).

The acute cognitive effects of cannabis use are well established, with impairments predominantly in the areas of attention, executive functioning, learning, and memory (Crean et al. 2011; Volkow et al. 2016; Zhornitsky et al. 2021). Residual effects of heavy cannabis use on cognition typically resolve within three weeks with sustained abstinence (Crean et al. 2011; Meier et al. 2018; Ross et al. 2020). However, some evidence supports persisting negative impacts on cognition for long-term, heavy users who initiate use during the neurodevelopmental period (age 25 and younger) and those with significant symptoms of cannabis use disorder (CUD) (Crean et al. 2011; Kroon et al. 2020; Lovell et al. 2020). Notably, the vast majority of cannabis research focuses on adolescent and young adults, leaving a significant gap in understanding its cognitive and neurobiological effects in older adults.

Emerging evidence suggests that the effects of cannabis on the aging brain may differ meaningfully from those observed in younger populations. Reviews focused on the cognitive effects of cannabis use in older adults, including those with cognitive decline and dementia, have concluded that low-dose, short-term medical cannabis use is generally well tolerated in older adults and does not confer significant risk for adverse cognitive outcomes (Scott et al. 2019; Vacaflor et al. 2020). Reviews incorporating both human and animal studies have identified potential neuroprotective properties of cannabis on cognition. For example, Pocuca et al. (2021) noted methodological limitations impacting the largely null effects of cannabis use on older adult cognition in human studies, while observing that better controlled animal models indicate that very low doses of THC improved cognition in very old rodents while slightly higher chronic doses of THC improved cognition in moderately aged rodents. Similarly, Weinstein and Sznitman (2020) also note important brain-age differences and emphasized the potential for cannabis to confer neuroprotective effects in older adults specifically. Relevant to older adult cognition across the dementia spectrum, some cannabinoids, including THC and CBD, have been associated with reduced neuroinflammation, enhanced cholinergic transmission, and reduced beta-amyloid aggregation, with exploratory application in human participants with Alzheimer’s disease (Abate et al. 2021; Aso & Ferrer, 2014; Bahji et al. 2020; Weinstein & Sznitman, 2020). These findings suggest differential effects of cannabis on cognition and brain health that vary by dose and age. However, rigorous controlled studies in older human populations remain limited.

Among older adults who reported any use of cannabis in the past year, subjective memory concerns (SMC) were endorsed at higher rates relative to non-users, with recent estimates ranging from 13.0% to 17.5%, compared to a significantly lower rate of 6.4% among non-users (Mulhauser et al. 2023; Vacaflor et al. 2020). Among a representative sample of US adults aged 50 years and older, 8.2% reported using cannabis in the past year, and use was associated with a two-fold increase in reporting SMC; however, this relationship was attenuated after accounting for demographic, health, and psychiatric factors, and the relationship was nonsignificant when evaluating past-month use of cannabis (Mulhauser et al. 2023). Even so, the literature on older adults who use cannabis remains comparatively small and is limited by several factors, including a lack of representation specific to older adults, limited information on cannabis use patterns among older adults, and minimal or no performance-based measures of neuropsychological functioning (Mulhauser et al. 2023; Scott et al. 2019; Volkow et al. 2016; Weinstein & Sznitman, 2020).

We sought to address these gaps in the literature by evaluating cognitive differences in older adults with and without recent cannabis use based on comprehensive neuropsychological test performance. Based on the literature indicating that low-dose cannabis use in older adults is generally well tolerated from a cognitive perspective, we hypothesized that older adults with recent cannabis use would demonstrate comparable neuropsychological test performance to non-users after accounting for relevant covariates. Furthermore, we explored how responses on the Cannabis Use Disorders Identification Test–Revised (CUDIT-R), a frequently used measure of cannabis-related problem severity and indicator of CUD, may be associated with cognitive outcomes.

## Method

### Participants and procedure

We extracted data from 588 participants spanning the dementia continuum from the University of Michigan Memory and Aging Project (UM-MAP) databank. UM-MAP is the primary longitudinal observational cohort of the Michigan Alzheimer’s Disease Center (MADC), which contributes common data elements to the National Alzheimer’s Coordinating Center (NACC) Uniform Data Set (UDS) (NACC, 2024). The first study visit in which participants reported their cannabis use history were included. UM-MAP participants were recruited from communities in southeastern Michigan, the neuropsychology and neurology clinics at the University of Michigan, the Healthier Black Elders Center through the Institute of Gerontology at Wayne State University, and the University of Michigan Health Research website. Inclusion criteria for participation in UM-MAP were aged 55 years or older, a research diagnosis consistent with NACC-defined criteria (see below), and accompaniment by a knowledgeable informant (e.g., spouse, partner, or child).

Prior to starting the evaluation, participants or their legally authorized representative provided informed consent. All human data included in this manuscript were obtained with approval from the University of Michigan Institutional Review Board (IRB). The study adhered to the ethical principles outlined in the Declaration of Helsinki. Study visits occur annually and consist of a standardized clinical evaluation comprised of demographic data, history and physical examination (including neurological examination), dementia staging, behavioral and functional assessments (including self- and informant-report questionnaires), and neuropsychological testing. Only data from the first time participants completed the cannabis questionnaire were included for the current analyses.

### Measures

#### UDS neuropsychological battery

The rationale, development, validation, harmonization, administration and scoring procedures, and cognitive status staging of the UDS neuropsychological battery are described in detail elsewhere (Dodge et al. 2020; Kiselica et al. 2020; Staffaroni et al. 2021). The UDS neuropsychological battery is derived from neuropsychological instruments and paradigms commonly used in clinical practice. The current version (UDSv3) consists of the following tests: Montreal Cognitive Assessment (MoCA), Craft Story 21 Recall, Benson Complex Figure, Number Span Test, Category Fluency, Phonemic Fluency, Trail Making Test A & B, and Multilingual Naming Test (MINT).

Prior factor analysis of the UDSv3 neuropsychological battery (Kiselica et al. 2020) identified a higher-order factor comprised of a supraordinate general cognitive factor and five lower-order factors: speed/executive (Trail Making Test A & B, Phonemic Fluency for both “F” and “L”), visual (Benson Complex Figure Copy and Recall trials), attention (Number Span Test forward and backward), language (Category Fluency for both animals and vegetables, MINT), and memory (Craft Story 21 Recall Immediate and Delayed). For the current study, raw scores for these measures were standardized into z-scores and then averaged to create composite *z*-scores for the five lower-order neuropsychological domains identified by Kiselica et al. (2020). As is common in clinical practice, the raw scores (seconds) for Trail Making Test parts A and B were truncated at 150 and 300 s, respectively. These two z-scores were also reversed to ensure that higher z-scores reflected better performance across all tests and composites.

#### Questionnaires

##### Cannabis use in the prior six months

Participants were asked, “Have you used any cannabis over the past six months?” Responses included “Yes” and “No.”

##### Cannabis Use Disorder Identification Test–Revised (CUDIT-R)

Participants who responded “Yes” to using cannabis in the past six months were then administered the CUDIT-R (Adamson et al. 2010), an 8-item self-report inventory that assesses behaviors and symptoms concerning for CUD over the past six months. Each item is scored on a 5-point scale ranging from 0 to 4, with higher scores reflecting greater frequency or severity of cannabis-related problems. Total scores range from 0 to 32; scores ranging from 8 to 11 indicate *hazardous cannabis use*, scores ≥ 12 indicate *possible cannabis use disorder*.

##### Geriatric Depression Scale–Short Form (GDS-SF)

The GDS-SF is a 15-item self-report inventory of depression symptoms commonly observed in older adults. Scores on the GDS-SF range from 0 to 15. Scores ≥ 5 indicate elevated levels of depression, while scores ≥ 9 warrant clinical attention and safety evaluation by a licensed mental health provider.

##### Covariates

Demographic information (i.e., age, sex, race, years of education) and clinical characteristics (i.e., depression, general cognitive functioning) were obtained from questionnaires and clinical interviews in accordance with NACC administrative procedures (see UDS references above). Alcohol and tobacco use were considered as potential covariates but ultimately excluded due to temporal and measurement inconsistencies. Per NACC protocol, these variables were recorded only at the initial study visit and carried forward across subsequent visits, which resulted in misalignment with the cannabis and cognitive data used in this study. Differences in measurement timeframes and formats (e.g., lifetime tobacco use, 3-month alcohol use, and 6-month cannabis use) further limited their appropriateness for inclusion in adjusted models.

### Data analytic plan

We screened the initial dataset to exclude participants who did not meet inclusion criteria. Participants with missing data on cognitive tests were excluded, as this precluded calculation of a domain composite score. There was no significant difference in the frequency distribution of cannabis use (users vs. non-users) by cognitive test data missingness (missing data vs. complete data), χ^2^(1, *N* = 588) = 0.29, *p* = .59.

We used multivariate, univariate, and propensity score matching (PSM) analyses to evaluate the associations between cannabis use and cognition in older adults. First, we used multivariate analysis of covariance (MANCOVA) to determine whether differences existed in cognitive performance by cannabis use (users vs. non-users). Age (years), sex (male vs. female), race (White, Black, Asian, Other), education (years), depression (GDS-SF total score), and global cognitive functioning (MoCA total score) were entered as covariates. The MoCA was included to account for diagnostic heterogeneity across participants, as our sample included individuals with normal cognition, MCI, and dementia. Although cannabis groups did not differ significantly in the distribution of diagnostic classifications, there was considerable variability in global cognitive functioning. Including the MoCA helped reduce confounding due to overall cognitive status while avoiding criterion contamination that would arise from using diagnosis – partially defined by the cognitive outcomes themselves – as a covariate. Assumptions of multivariate normality and homogeneity of variance–covariance matrices were tested using Mardia’s test of kurtosis (α = 0.05) and Box’s *M* (α = 0.05), respectively. Multivariate outliers were identified using Mahalanobis distance (α = 0.05). A significant omnibus MANCOVA was followed by five multivariable linear regressions for each cognitive domain. All significance tests were two-tailed with α = 0.05.

To better balance the groups and control for potential confounding variables, we employed PSM using a 2:1 nearest-neighbor matching strategy (with replacement) between non-users and users of cannabis as a sensitivity analysis (Austin, 2010). Specifically, cannabis users and non-users were matched based on the propensity scores (i.e., probability of cannabis use) based age (years), sex (male vs. female), race (White vs. Black), education (years), depression (GDS-SF total score), and global cognitive functioning (MoCA total score). We excluded participants who identified as Asian or Other race due to small cell sizes that precluded matching. After matching, we estimated the Average Treatment Effect on the Treated (ATT) to compare cognitive performance between the matched groups across the same five cognitive domains.

Finally, on an exploratory basis, we examined whether cannabis-related problem severity was associated with cognitive performance among participants who reported recent cannabis use. Participants were categorized using validated CUDIT-R total score cutoffs as described above. We conducted analyses of covariance (ANCOVAs) across the five UDSv3 cognitive domains, retaining the same covariates from prior models. In a secondary exploratory analysis, we assessed bivariate correlations between individual CUDIT-R items and each cognitive domain score to identify potential item-level associations.

## Results

### Descriptive statistics

Descriptive statistics for the total sample and by cannabis use appear in Table 1. Ages ranged from 55 to 93 years (*M* = 72.52, *SD* = 6.45). Biological sex was distributed as 195 (36.11%) male and 345 (63.39%) female. Most participants identified as White (*n* = 366, 67.78%), with approximately one-third identifying as Black or African American (*n* = 166, 30.74%). Years of education ranged from 8 to 21 and was truncated at 20 years (*M* = 16.33, *SD* = 2.37). Most participants were right-handed (*n* = 462, 89.71%). Any cannabis use in the prior 6 months was self-reported as either “yes” (*n* = 60, 11.11%) or “no” (*n* = 480, 88.93%). MoCA scores ranged from 11 to 30 (*M* = 25.23, *SD* = 3.53). Using χ^2^ tests of independence, cannabis use was not significantly associated with sex, race, handedness, or diagnostic classification (*p*’s > .05). When using independent-samples *t*-tests or the Mann-Whitney *U* test, years of education, number of medical conditions, and MoCA scores were not significantly different between users and non-users of cannabis (*p*’s > .05). Relative to non-users of cannabis (*M* = 72.81, *SD* = 6.59), the cannabis users were younger (*M* = 70.22, *SD* = 4.61; *p* ≤ .001). Cannabis users scored higher (*M* = 2.07, *SD* = 2.25) than non-users (*M* = 1.32, *SD* = 1.82) on a screening measure of depression (GDS-SF; *p* = .02).

**Table 1.** Demographic and clinical characteristics (N = 540)

Variable	Total	Cannabis +	Cannabis –	χ^2^ / *t*	*p*
*N* (%)	540 (100)	60 (11.11)	480 (88.89)	—	
Age, *M* (*SD*)	72.52 (6.45)	70.22 (4.61)	72.81 (6.59)	3.88	≤.001
Education, *M* (*SD*)	16.33 (2.37)	16.42 (2.40)	16.32 (2.37)	−0.31	.76
Sex				2.31	.13
Male	195 (36.11)	27 (45.00)	168 (35.00)		
Female	345 (63.39)	33 (55.00)	312 (65.00)		
Race				2.14	.54
White	366 (67.78)	41 (68.33)	325 (67.71)		
Black/African American	166 (30.74)	18 (30.00)	148 (30.83)		
Asian	5 (0.93)	—	5 (1.04)		
Other	3 (0.56)	1 (1.67)	2 (0.42)		
Handedness (*n* = 515)				1.08	.58
Right	462 (89.71)	50 (82.29)	412 (89.76)		
Left	46 (8.93)	6 (10.71)	40 (8.71)		
Ambidextrous	7 (1.36)	—	7 (1.53)		
# Medical Conditions, *M* (*SD*^a^)	2.74 (1.56)	2.68 (1.45)	2.75 (1.58)	0.30	.76
GDS-SF, *M* (*SD*)	1.40 (1.89)	2.07 (2.25)	1.32 (1.82)	−2.49	.02
MoCA, *M* (*SD*)	25.23 (3.53)	26.00 (3.03)	25.13 (3.58)	−1.81	.07
Diagnostic Classification				2.19	.34
Normal	321 (59.44)	39 (65.00)	282 (58.75)		
MCI	176 (32.59)	19 (31.67)	157 (32.71)		
Dementia	43 (7.96)	2 (3.33)	41 (8.54)		

*Note.* GDS-SF, Geriatric Depression Scale-Short Form; MCI, Mild Cognitive Impairment. MoCA, Montreal Cognitive Assessment. Age, education, and number of medical conditions are presented as means and standard deviations. Differences in the means or frequencies by cannabis use (users *vs*. non-users) are reported as Welch’s *t*-tests and Chi-squared (χ^2^) tests of independence, respectively.

a The Mann-Whitney *U* Test was utilized to determine whether differences existed in the number of medical conditions by cannabis use status (*n* = 516).

### Inferential statistics

#### Group differences by recent use of cannabis

##### Initial Model

The initial omnibus MANCOVA was statistically significant, Wilks’ Λ = 0.31, *F*(45, 2356) = 15.50, *p* ≤ .001. The multivariate *R* ^2^ was .69. Box’s *M* supported homogeneity of variance–covariance matrices (*p* = .19). However, Mardia’s test of kurtosis (*p* ≤ .001) for the residuals suggested departure from a multivariate normal distribution. Examination of Mahalanobis distance revealed 18 outliers that exceeded the χ^2^ critical value (12.83) for *df* = 5 dependent variables and α = .05. Given the impact of these violations on MANCOVA, these 18 participants (1 cannabis user, 17 non-users) were excluded from this analysis, yielding an analytic sample of 522.

##### Final Model

Table 2 depicts the MANCOVA results with *N* = 522. The omnibus MANCOVA was again statistically significant, Wilks’ Λ = 0.32, *F*(45, 2275) = 14.83, *p* ≤ .001. The multivariate *R* ^2^ was .68. At the multivariate level, age (*p* ≤ .001; multivariate *R* ^2^ = .05), MoCA total score (*p* ≤ .001; multivariate *R* ^2^ = .54), sex (*p* = .01; multivariate *R* ^2^ = .03), and race (*p* ≤ .001; multivariate *R* ^2^ = .12) were significant covariates. Cannabis use was non-significant (*p* = .30; multivariate *R* ^2^ = .01). Mardia’s test for multivariate kurtosis (*p* = .92) and Box’s *M* (*p* = .87) supported multivariate normality and homogeneity of variance–covariance matrices, respectively .

**Table 2.** Multivariate analysis of covariance (MANCOVA) of five neuropsychological domains by cannabis use and relevant covariates (N = 522)

Independent Variable	Wilks’ Λ	*F*(5, 508)	*p*
Cannabis Use	0.99	1.22	0.30
Age	0.95	4.87	≤0.001
GDS-SF	0.98	1.69	0.13
MoCA	0.46	118.41	≤0.001
Education	0.99	1.37	0.23
Sex	0.97	3.04	0.01
Race	0.88	4.27	≤.001

*Note.* GDS-SF, Geriatric Depression Scale-Short Form. MoCA, Montreal Cognitive Assessment. Sex was coded as 1 = male, 2 = female. Race was coded as 1 = White, 2 = Black, 5 = Asian, and 50 = Other. The overall multivariate analysis of covariance (MANCOVA) was statistically significant, Wilks’ Λ = 0.32, *F*(45, 2275) = 14.83, *p* ≤ .001, Multivariate *R* ^2^ = .68. The dependent variables included five neuropsychological domain composite scores: speed/executive functioning, visual, attention, language, and memory.

Given the significant omnibus MANCOVA, five univariate multiple regression models followed (Table 3). Omnibus tests for these five regressions indicated statistically significant models (*p* ≤ .001) for all five cognitive domains: speed/executive, visual, attention, language, and memory. Regarding covariates, overall cognitive functioning – as measured by the MoCA total score – was a statistically significant positive predictor in all five regression models.

**Table 3.** Univariate multivariable linear regression of five neuropsychological domains by cannabis use, age, substance use, and depression (N = 522)

Predictor	Speed/Executive	Visual	Attention	Language	Memory
	*B* (*SE*)	*B* (*SE*)	*B* (*SE*)	*B* (*SE*)	*B* (*SE*)
Cannabis Use					
No (ref.)	—	—	—	—	—
Yes	0.05 (0.07)	−0.09 (0.08)	−0.07 (0.11)	0.10 (0.08)	0.17 (0.10)
Age	−0.01 (0.01)***	−0.01 (0.01)*	0.01 (0.01)	−0.01 (0.01)*	−0.01 (0.01)
GDS	−0.02 (0.01)	−0.01 (0.01)	0.01 (0.02)	−0.01 (0.01)	−0.03 (0.02)
MoCA	0.10 (0.01)***	0.11 (0.01)***	0.09 (0.01)***	0.11 (0.01)***	0.16 (0.01)***
Education	0.01 (0.01)	0.01 (0.01)	0.03 (0.02)	0.01 (0.01)	−0.01 (0.01)
Sex	0.13 (0.05)**	−0.05 (0.06)	0.01 (0.08)	0.16 (0.05)**	0.07 (0.07)
Race					
White (ref.)	—	—	—	—	—
Black	−0.28 (0.05)***	0.08 (0.06)	−0.27 (0.08)**	−0.28 (0.05)***	0.01 (0.07)
Asian	0.10 (0.22)	0.27 (0.26)	−0.11 (0.35)	−0.21 (0.24)	−0.05 (0.33)
Other	0.51 (0.29)	0.52 (0.33)	0.77 (0.45)	−0.03 (0.31)	−0.18 (0.42)
Constant	−1.63 (0.35)	−1.96 (0.40)	−2.76 (0.54)	−2.13 (0.37)	3.22 (0.50)
*F*(9, 512)	42.78	24.85	12.37	41.45	34.60
*R* ^2^	.43***	.30***	.18***	.42***	.38***

*Note.* Non-use of cannabis was entered as the reference category. The five outcome variables for the univariate models were speed/EF, visual, attention, language, and memory.

^+^ *p* < .10. *p < .05. **p < .01. ***p < .001.

Regarding cannabis use (users vs. non-users), estimated means with standard errors for the five regression models are presented in Table 4. The means are adjusted for covariates (i.e., age, sex, race, years of education, depression, MoCA total score). The differences in *z*-scores between users and non-users of cannabis ranged from -0.09 to 0.17. None of the tests of differences reached statistical significance (*ps* > .11). That is, there was no significant difference in cognitive domain performance between users and non-users of cannabis after controlling for relevant covariates.

**Table 4.** Estimated means and standard errors for five neuropsychological domains by cannabis use in the multivariable linear regression (N = 522)

Domain	Cannabis use		Difference	*t*	*p*
	Yes	No			
Speed/Executive	0.15 (0.07)	0.11 (0.02)	0.05	0.66	.51
Visual	0.03 (0.08)	0.12 (0.03)	−0.09	−1.06	.29
Attention	0.01 (0.10)	0.08 (0.04)	−0.07	−0.62	.53
Language	0.11 (0.02)	0.21 (0.07)	0.10	1.27	.20
Memory	0.26 (0.10)	0.10 (0.03)	0.17	1.62	.11

*Note.* The estimated means and standard errors for the five neuropsychological domains are represented as *z*-scores (*M* = 0, *SD* = 1). Means are adjusted for covariates in the univariate multiple regression model: age, sex, race, education, depression (Geriatric Depression Scale-Short Form), and global cognitive functioning (Montreal Cognitive Assessment [MoCA] score).

#### Propensity score matching

PSM was used to estimate mean differences in performance across five cognitive domains between recent cannabis users (*n* = 59) and non-users (*n* = 93). PSM controls for confounding variables by matching participants based on their likelihood (propensity) of cannabis use, calculated using covariates including age, sex, race, education, depression score, and global cognitive functioning (MoCA score). Matching was performed using a 2:1 nearest-neighbor algorithm with replacement, meaning that non-users could be matched to more than one cannabis user if they had a similar propensity score. This approach ensures that comparisons are made between individuals with equivalent likelihoods of cannabis use, rather than requiring direct, one-to-one matches between participants. The analyses revealed no significant differences between cannabis users and non-users in any cognitive domain (all p’s > .38; see Table 5).

**Table 5.** Propensity score matching on five cognitive domains by cannabis use

Outcome	*N*	Cannabis +	*N*	Cannabis -	*t*	*p*	*d*
Speed/Executive	59	0.11	93	0.10	0.05	.96	0.002
Visual	59	0.12	93	0.21	−0.83	.41	0.067
Attention	59	0.08	93	0.12	−0.30	.76	0.155
Language	59	0.28	93	0.20	0.61	.54	−0.139
Memory	59	0.35	93	0.22	0.88	.38	−0.12

*Note.* Differences in the neuropsychological domain performances by cannabis use (users vs. non-users) are estimated using propensity score matching. Cannabis users were matched to non-users in 2:1 nearest-neighbor matching strategy (with replacement) by age, sex, race, education, depression score (Geriatric Depression Scale-Short Form), and global cognitive functioning (Montreal Cognitive Assessment [MoCA] score).

*p* < .05. Means are presented as *z*-scores (*M* = 0, *SD* = 1).

#### Cannabis-related problem severity and cognitive performance

To further explore the relationship between cannabis use and cognition, we conducted follow-up analyses using participants’ total scores on the CUDIT-R. Based on established cutoffs, we initially categorized participants into three groups: “within normal limits” (total score of 0–7; *n* = 45), “hazardous use” (total score of 8–11; *n* = 11), and “cannabis use disorder” (total score of 12–32; *n* = 4). Due to small cell sizes, the hazardous use and cannabis use disorder groups were combined, resulting in a dichotomous classification: within normal limits (*n* = 45, 75%) and hazardous/problematic use (*n* = 15, 25%). Notably, two participants who endorsed cannabis use in the past six months scored zero on all eight CUDIT-R items, suggesting minimal engagement beyond a single use.

We conducted a series of ANCOVAs to evaluate whether this dichotomous indicator of cannabis use severity was associated with cognitive performance, controlling for age, sex, race, education, GDS total score, and MoCA total score. There were no significant differences between groups for speed/executive, attention, visual, or language domains (all *ps* > .28). However, a significant small-to-moderate effect was observed for memory performance, *F*(1, 51) = 6.66, *p* = .01, *R* ^*2*^ = .12. Estimated marginal means indicated that participants scoring within normal limits on the CUDIT-R performed significantly better on memory tasks (M = 0.53, SE = 0.12) than those with hazardous/problematic cannabis use (M = −0.17, SE = 0.22). See Table 6 for details. This effect remained significant after removing the two participants with CUDIT-R scores of 0, *F* (1, 49) = 5.77, *p* = .02, with estimated means of *M* = 0.50 (SE = 0.12) and M = −0.15 (SE = 0.23), respectively.

#### Exploratory correlations between CUDIT-R items and cognitive domains

To further explore the relationship between cannabis-related behaviors and cognition, we examined correlations between individual CUDIT-R items and the five cognitive domains. Item 3 (“How often during the past 6 months did you find that you were not able to stop using cannabis once you had started?”) was negatively correlated with performance in four cognitive domains: speed/executive (r = −0.29, *p* = .024), visual (r = −0.44, *p* < .001), language (r = −0.28, *p* = .033), and memory (r = −0.30, *p* = .022). In addition, memory performance was significantly negatively correlated with Item 1 (frequency of use; r = −0.35, *p* = .006) and Item 6 (“How often… have you had a problem with your memory or concentration after using cannabis?”; r = −0.43, *p* < .001) See Table 7 for details.

**Table 7.** Correlations between CUDIT-R items and cognitive domains (N = 60)

CUDIT-R Item	Speed/Executive	Visual	Attention	Language	Memory
1. Frequency	−0.069	−0.079	−0.175	−0.058	−0.350**
2. Hours stoned	−0.205	−0.117	−0.032	−0.048	−0.096
3. Difficulty stopping	−0.292*	−0.439**	−0.158	−0.275*	−0.295*
4. Time spent	0.073	−0.004	−0.091	0.078	−0.146
5. Neglected roles	−0.252	−0.227	−0.236	−0.118	−0.098
6. Memory/concentration	−0.175	0.015	−0.082	−0.042	−0.428**
7. Others concerned	−0.056	0.060	−0.148	−0.017	−0.197
8. Tried to cut down	−0.193	0.208	−0.049	−0.014	−0.051

*Note.* *p < .05. **p < .01.

## Discussion

Overall, our results indicate that older adults who reported using cannabis in the prior six months did not differ on neuropsychological testing results when compared to older adults who reported no recent use of cannabis. This pattern held true across both traditional analytical approaches (multivariate and univariate regressions) and propensity score matching, which was employed to balance groups and enhance causal inference. Participants who used cannabis and scored in the hazardous/problematic use range on the CUDIT-R demonstrated lower performance in the memory domain compared to participants who used cannabis but scored within normal limits on the CUDIT-R. Exploratory associations between item level data from the CUIT-R and cognitive outcomes suggest that difficulty stopping cannabis use (CUDIT-R Item 3) may be associated with broader cognitive difficulties across domains (i.e., speed/executive, visual, language, and memory).

Our results are generally consistent with findings from systematic reviews concluding that low frequency cannabis use among older adults is generally well tolerated and is not clearly associated with adverse cognitive outcomes (Scott et al. 2019; Vacaflor et al. 2020; Weinstein & Sznitman, 2020). Yet, these findings stand in contrast to increased rates of SMC in older adults who use cannabis (Mulhauser et al. 2023; Vacaflor et al. 2020). While SMC is common in older adults and frequently reported by individuals who perform within normal limits on comprehensive neuropsychological evaluation (Crumley et al. 2014), SMC has also been shown to be a harbinger of cognitive decline and future diagnosis of MCI and dementia (Lee & Foster, 2023). Therefore, clarifying the relationship between increased SMC but generally similar objective cognition in older adults who use cannabis is worth further investigation. It remains a commonly held perception that cannabis use of any kind is deleterious to cognition (Carliner et al. 2017), and this perception may contribute to increased concern for SMC among older adults who use cannabis. As older adults and their medical providers are increasingly considering cannabis to manage chronic health conditions, additional data on the interactive effects across cannabis use patterns, aging trajectories, co-occurring medical conditions, and cognitive status will help inform treatment decisions and manage potential risks.

Scott et al. (2019) reviewed 26 studies examining cannabis use and cognitive outcomes in older adults, including participants with normal aging, neurodegenerative diseases, and common medical conditions. Overall, they concluded that “higher doses and heavier use of cannabis are associated with modest negative effects” on cognition, while also noting that “the cognitive deficits associated with heavy, recreational cannabis may not be applicable to medical cannabis users, who may use products with less THC and experience relief from other symptoms, which may contribute to improved cognitive functioning” (Scott et al. 2019, p. 452). Our study found no domain-level group difference in cognition (negative or positive) between older adults with and without recent cannabis use. Potentially consistent with Scott et al. (2019), our subsample of cannabis users who endorsed elevated risk for CUD demonstrated lower memory performance when compared to cannabis users without increased risk for CUD. The frequency of cannabis use in our sample ranged from *less than monthly* (41%) to *4 or more times per week* (24%) with a median response of *2-4 times per month*. Like Scott et al. (2019), our sample also lacks clear information on relevant use patterns that may inform outcomes. Even so, our study addresses some limitations identified by Scott et al. (2019) by using comprehensive performance-based neuropsychological testing results in the evaluation of older adults with mixed cognitive status.

In a secondary exploratory analysis, individual CUDIT-R items showed modest associations with cognitive performance, particularly within the memory domain. Notably, self-reported difficulty stopping cannabis use (CUDIT-R Item 3) was associated with lower performance across multiple cognitive domains (i.e., speed/executive, visual, language, memory), suggesting that diminished behavioral regulation around cannabis use may reflect broader cognitive vulnerabilities. In contrast, items reflecting frequency of use and cannabis-related memory complaints were more selectively associated with lower memory performance. These findings are consistent with recent work by Anquillare et al. (2023), who found that greater cannabis-related problem severity was linked to higher self-reported cognitive failures and reduced cognitive self-efficacy in older adults. Although preliminary, our findings suggest that measurable cognitive differences may be linked to problematic patterns of cannabis use (i.e., impaired control, cannabis-related cognitive concerns), whereas such deficits are not observed in older adults who use cannabis without symptoms of a use disorder.

Several considerations and limitations are important when interpreting our results. First, like many studies of older adult cannabis use and cognition, several salient details about cannabis use patterns in our sample are unknown. For example, this sample does not include data on historical use patterns (e.g., age of onset, history of substance use disorder), motivational factors (e.g., recreational vs. medical), cannabinoid content (e.g., relative THC vs. CBD), or routes of administration (e.g., gummies, joints, tinctures), each of which may inform the associations between cannabis use and cognitive functioning in older adults. Second, our sample of older adults reporting recent cannabis use is small. Even so, this longitudinal cohort continues with recruitment and annual neuropsychological evaluations. As cannabis-related questions are now part of ongoing data collection and rates of cannabis use continue to rise among older adults, future studies will be positioned to examine within-person changes in cannabis use over time and their potential association with cognitive trajectories. Such longitudinal analyses will provide a more nuanced understanding of the dynamic relationship between cannabis use patterns and cognitive aging. Appropriate data on other substance use variables were not available for inclusion as covariates due to significant variability in data collection timing and response formats in the dataset; future studies will benefit from more robust and standardized assessment of behavioral use patterns across substances. Finally, we were not able to evaluate differential outcomes based on cognitive status or neurocognitive diagnosis due to the currently small sample of those reporting use. Further analysis of differences in cognition according to recent cannabis use within each cognitive phenotype may reveal subgroups that experience different risk/benefit profiles. Despite these limitations, this study advances our currently limited understanding of cannabis use and cognition in older adults by addressing current gaps in the literature and identifying areas for future research.

## Conclusions

Our study contributes to the literature on cannabis use and cognition in older adults by examining comprehensive neuropsychological outcomes associated with recent (i.e., past six months) cannabis use in a well-characterized sample of older adults spanning the dementia continuum. Across the entire group, cognitive functioning was similar between older adults with and without recent cannabis use across five domains of cognition. Among older adults who reported recent use of cannabis, scoring in the hazardous/problematic use range on the CUDIT-R was associated with lower performance on memory tasks. Future research should incorporate detailed assessment of cannabis use patterns and individual characteristics to better understand the nuances of these relationships and potential differences based on cognitive phenotype.

**Table 6.** F-ratios from analyses of covariance (ANCOVAs) for cannabis use and cognitive functioning (N = 60)

Variable	Speed/Executive	Visual	Attention	Language	Memory
Cannabis use problems^a^	0.50	0.03	1.20	0.03	6.66*
Age (y)	0.36	1.02	0.00	4.71*	2.05
Sex	2.75	0.60	0.02	1.15	0.05
Race	2.09	1.01	2.05	8.05***	0.57
Education (y)	0.34	0.43	0.35	1.47	0.53
GDS-SF	1.76	2.84	0.28	0.18	0.18
MoCA	35.76***	9.94**	3.46	14.88***	4.14*
*R* ^2^	.60***	.29*	.17	.54***	.31*

*Note.* GDS, Geriatric Depression Scale-Short Form. MoCA, Montreal Cognitive Assessment. Age, sex (male vs. female), race (White, Black, Other), years of education, depression (GDS-SF), and general cognitive functioning (MoCA) were included in all five ANCOVAs as relevant covariates. The values depicted represent the *F*-ratios in the ANCOVAs.

a Cannabis use disorder was measured as a dichotomous variable (0 = *within normal limits*, 1 = *hazardous/problematic use*) using established cutoff values from the Cannabis Use Disorder Identification Test (CUDIT).

*p* < .05. **p < .01. ***p < .001.
